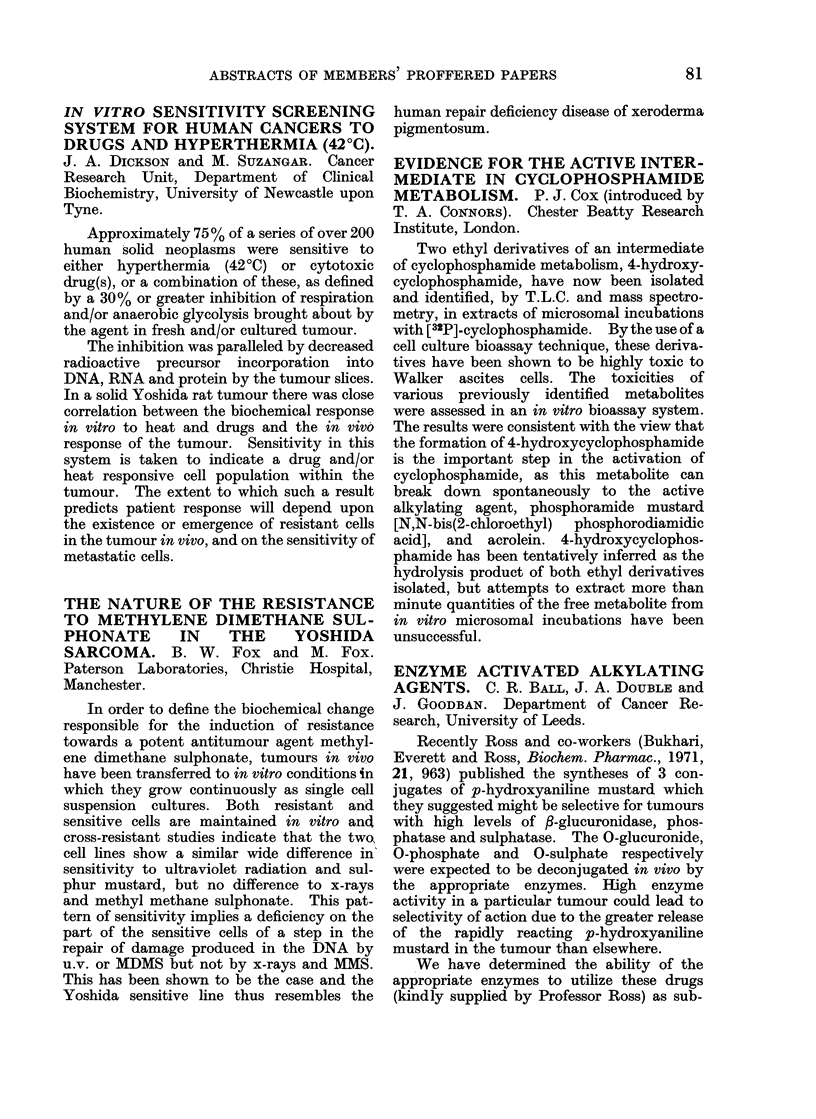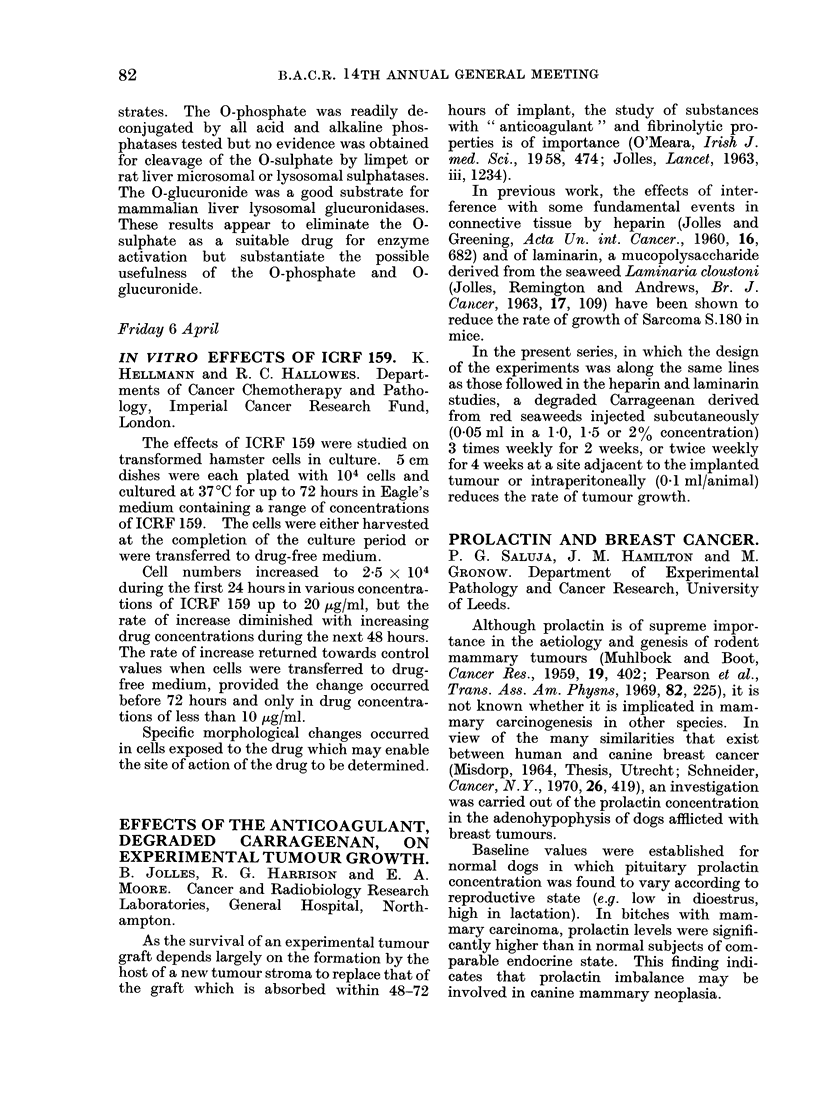# Enzyme activated alkylating agents.

**DOI:** 10.1038/bjc.1973.92

**Published:** 1973-07

**Authors:** C. R. Ball, J. A. Double, J. Goodban


**ENZYME ACTIVATED ALKYLATING AGENTS.** C. R. BALL, J. A. DOUBLE and J. GOODBAN. Department of Cancer Research, University of Leeds.

Recently Ross and co-workers (Bukhari, Everett and Ross, *Biochem. Pharmac.*, 1971, **21**, 963) published the syntheses of 3 conjugates of *p*-hydroxyaniline mustard which they suggested might be selective for tumours with high levels of β-glucuronidase, phosphatase and sulphatase. The O-glucuronide, O-phosphate and O-sulphate respectively were expected to be deconjugated *in vivo* by the appropriate enzymes. High enzyme activity in a particular tumour could lead to selectivity of action due to the greater release of the rapidly reacting *p*-hydroxyaniline mustard in the tumour than elsewhere.

We have determined the ability of the appropriate enzymes to utilize these drugs (kindly supplied by Professor Ross) as substrates. The O-phosphate was readily deconjugated by all acid and alkaline phosphatases tested but no evidence was obtained for cleavage of the O-sulphate by limpet or rat liver microsomal or lysosomal sulphatases. The O-glucuronide was a good substrate for mammalian liver lysosomal glucuronidases. These results appear to eliminate the O-sulphate as a suitable drug for enzyme activation but substantiate the possible usefulness of the O-phosphate and O-glucuronide.